# Modeling and Validation of Material Removal Based on Rheological Behavior Under Dynamic-Viscosity Nonlinear Coupling Effects

**DOI:** 10.3390/mi16050572

**Published:** 2025-05-13

**Authors:** Tianchen Zhao, Luguang Guo, Qilong Gao, Xu Wang, Binghai Lyu, Chen Li

**Affiliations:** 1 College of Mechanical Engineering, Quzhou University, Quzhou 324000, China; zhaotianchen1989@126.com; 2 College of Mechanical Engineering, Zhejiang University of Technology, Hangzhou 310023, China; gao_qilong@163.com (Q.G.); wx382935877@163.com (X.W.); icewater7812@126.com (B.L.); 3 School of Mechatronics Engineering, Harbin Institute of Technology, Harbin 150001, China; hit_chenli@163.com

**Keywords:** non-Newtonian fluid, fluid dynamics, tribology principle, material removal

## Abstract

Compliant rheological polishing advanced in facilitating the generation of smooth curved surfaces. However, the inherent energy dissipation of the medium during flow results in an uncontrollable material removal distribution. This study proposes utilizing the motion of the tool to regulate the distribution of physical fields within the computational domain, thereby controlling material removal. A film thickness model is developed based on fluid dynamics and tribology principles to examine the pressure and velocity distributions within the film. In conjunction with contact mechanics and metallography, a material removal model is formulated and then validated and refined by valid experiment, demonstrating a positive correlation between material removal rate and surface quality. Optimization experiments produced a curved surface with an *Ra* of 17.59 nm.

## 1. Introduction

Compliant fluid polishing technologies, such as Magnetorheological Finishing (MRF) and Shear-Thickening Polishing (STP), have garnered significant attention in recent years as non-traditional processing methods for high-precision surface machining [1,2,3,4,5]. Compared to traditional ultra-precision machining technology, these methods leverage the flow characteristics of liquid media to conform to the workpiece surface, enabling efficient material removal and facilitating the processing of complex curved surfaces and microstructures [6,7,8,9,10,11,12]. They are widely used in the final machining of optical components [13,14], aerospace components [15,16] and semiconductor devices [17,18].

However, due to the complex flow dynamics and shear stress distribution inherent in fluids, the material removal rate often exhibits substantial uncertainty [19], presenting a significant challenge for achieving high-precision surface machining. In compliant fluid polishing, the material removal distribution is primarily influenced by factors such as fluid viscosity, shear rate, abrasive concentration, and flow field distribution [20,21,22]. The nonlinear coupling of these factors makes it difficult to predict material removal distribution during polishing, complicating efforts to control uniformity in material removal.

Peng et al. analyzed the mechanism of inlet pressure drop during abrasive flow machining (AFM) processing using capillary flow and developed a constitutive model for high shear rate medium [23]. Kum et al. applied AFM to polish nozzle guide vanes, establishing a baseline for material removal distribution, which was later compensated by design modifications, reducing dimensional errors from 600 μm to 200 μm [24]. Building on this approach, Fu and Guo used AFM and STP to polish blades and bearing raceways, respectively, achieving high-precision material removal while maintaining excellent surface quality. After 400 cycles, Fu achieved a surface roughness *Ra* of 0.141 μm, and the standard deviation decreased from 0.0977 μm (without compensation) to 0.0291 μm [25]. Guo obtained a surface roughness of *Ra* 11.16 nm with a variance of 0.58 nm^2^ after 90 min of polishing. Ke et al. employed shear thickening polishing (STP) to passivate cemented carbide inserts, achieving a reduction in cutting edge roughness from 118.01 nm to 8.13 nm within 10 min [26].

However, the compensation conditions for this approach are highly dependent on the workpiece geometry. Once the workpiece undergoes modification, a complete re-experimentation of the process is necessary, which limits the feasibility of accurately predicting the process. MRF provides a viable alternative: by calculating the relationship between the physical field distribution in the computational domain and the motion of the polishing tool, thereby enabling controlled material removal through carefully planned tool paths [27,28,29]. Zhu used a bonnet as the polishing tool and shear-thickening slurry as the medium, achieving a sub-micron bi-sinusoidal surface with a form error deviation of just 95 nm P–V [30,31]. Some researchers proposed magnetorheological fluid-assisted AFM methods, but these methods are essentially extensions of magnetorheological polishing and do not offer innovations to the underlying process [32,33]. Abrasive liquid jet polishing uses a jet of abrasive medium as a special polishing tool, which can also achieve high-precision material removal. However, its low processing efficiency, high nozzle wear, and rapid energy attenuation restrict further development of the technology [34,35].

This study examines the challenge of achieving deterministic material removal in compliant fluid polishing technologies, with a particular focus on shear-thickening fluids. It explores the physical field distribution within the computational domain of the polishing fluid assisted by the polishing tool and investigates the dynamic coupling mechanism between pressure and viscosity. By integrating experimental and numerical simulation methods, the results offer theoretical foundations and technical support for achieving high-precision control in compliant fluid polishing.

## 2. Principle of Dynamic-Viscous Shear-Thickening Polishing

Analysis of the shear thickening mechanism indicates that effective shear thickening polishing (STP) relies on generating a sufficiently high velocity gradient (shear rate) at the workpiece surface. However, under certain motion conditions, the high viscosity of the fluid leads to minimal slip between the slurry and the surface, resulting in negligible material removal even under high pressure. Additionally, the distribution of material removal is highly non-uniform.

To address this, a flexible tool movement is proposed to assist in STP processing. This movement actively creates a fluid shear zone at the workpiece surface, leading to localized rapid pressure and shear thickening. By leveraging the shear thickening effect of the slurry and its combined dynamic pressure lubrication effect, a high-viscosity, high-pressure polishing film is formed on the workpiece surface. This polishing film moves with the tool, generating relative motion between the tool and workpiece, thereby enabling ultra-precision processing of the surface.

**Figure 1:**
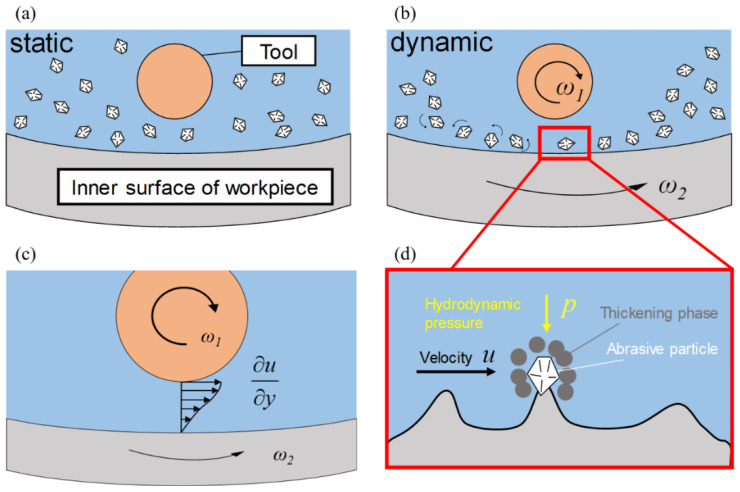
Principle of STP based on the dynamic-viscous pressure effect: (**a**) the slurry is static, (**b**) the slurry moves with the tool’s rotation, (**c**) distribution of the velocity gradient, and (**d**) schematic of the force acting on the abrasives.

The specific processing procedure is illustrated in Figure 1. The workpiece and polishing tool are immersed in the slurry. When the workpiece and tool are stationary in the fluid, the thickening phase exerts a lifting effect, causing the abrasive particles to be evenly dispersed in the slurry (Figure 1a). At this stage, there is no relative motion between the abrasive particles and the workpiece. As relative motion occurs between the workpiece and tool (Figure 1b), a velocity gradient is formed between them (Figure 1c). The shear thickening effect of the slurry causes a rapid increase in viscosity, and the thickening phase encapsulates the abrasive particles, driving them to scratch the workpiece surface and remove material. Figure 1d shows the force diagram of a single abrasive particle. The dynamic pressure p exerted on the particle is positively correlated with viscosity and velocity difference. High shear rates result in larger normal pressures and higher viscosities, which further increase the normal pressure. Under the combined action of these factors, the dynamic pressure p exerted by the polishing film is significantly higher than the p produced by the Newtonian slurry, thus enabling efficient material removal.

This method takes into account the coupling effect between pressure and viscosity, and is therefore referred to as the dynamic-viscous non-Newtonian polishing method (DVNNP). When the slurry is a shear-thickening fluid, this effect contributes to improved material removal. However, when the slurry is a shear-thinning fluid, the effectiveness should be evaluated based on the specific experimental conditions, comparing the influence of various factors on the magnitude of material removal.

**Figure 2:**
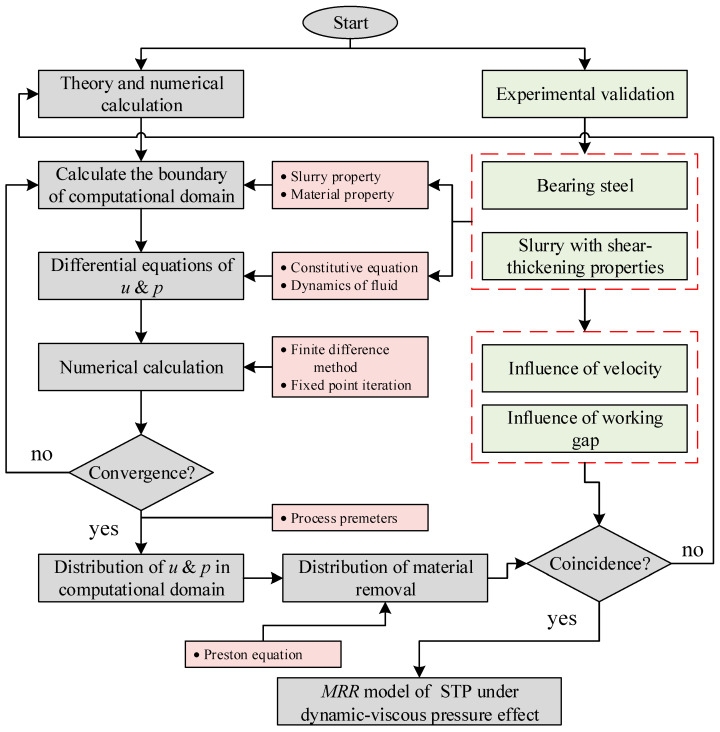
Flowchart of the research approach to material removal.

Figure 2 illustrates the research approach in this paper. First, the processing model was simplified using Taylor expansion to define the effective processing area. The pressure and velocity partial differential equations were derived from the film lubrication equation, and numerical solutions were obtained using the Euler method. Material removal distribution was determined through metallographic analysis and contact mechanics and compared with experimental results for correction. The final model represents the material removal in non-Newtonian fluid polishing under dynamic-viscous pressure effects.

### 2.1. Assumptions

To ensure the logical rigor of the theoretical derivation, the following reasonable basic assumptions are necessary [36]:

(1) The dispersive phase and abrasives are uniformly distributed in the liquid. The transition from one control point to another is continuous, and the analysis can be conducted using infinitesimal methods. Discontinuities only occur at the boundaries, consistent with the continuous medium model.
(2) The properties of the material (including viscosity) may vary spatially, but such changes occur gradually, reflected in the spatial dependence of material properties in the continuous medium theory equations.
(3) Viscous forces dominate, while inertial forces can be neglected. Additionally, there is no relative motion between the thickened and solidified dispersive phase and the abrasives.
(4) The surface tension of the slurry is neglected, and due to the relatively short processing time in this experiment, temperature rise caused by friction and the evaporation of the liquid are ignored (according to a previous study) [37].
(5) The slurry is incompressible and isotropic; thus, the computational fluid dynamics is based on pressure solutions.
(6) Due to the high viscosity and the small thickness of the polishing film, pressure variation along the thickness direction is negligible.

All the analyses and conclusions in this paper are based on these assumptions.

### 2.2. Rheological Performance

**Figure 3:**
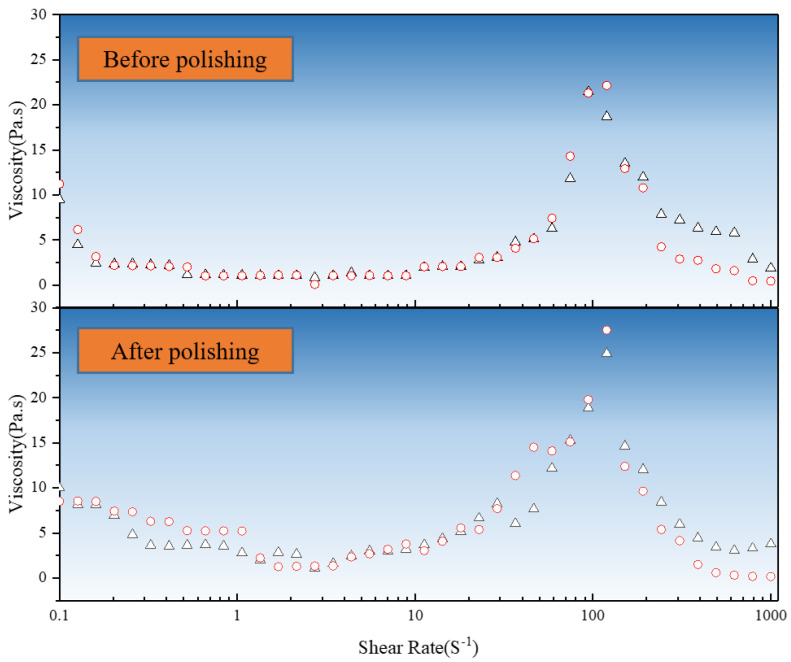
Comparison of rheological properties before and after polishing.

To assess the performance retention of the slurry during the processing time, rheological curves before and after processing were measured for two sets. As shown in Figure 3, the rheological curves in the thickening region follow a power-law behavior, and the rheological performance is stable before and after processing.

The fitted constitutive equation is given by the following equation:

(1) $$\tau =\mu {\dot{\gamma }}^{n}=0.008{\dot{\gamma }}^{2.6}$$

### 2.3. Machining System

**Figure 4:**
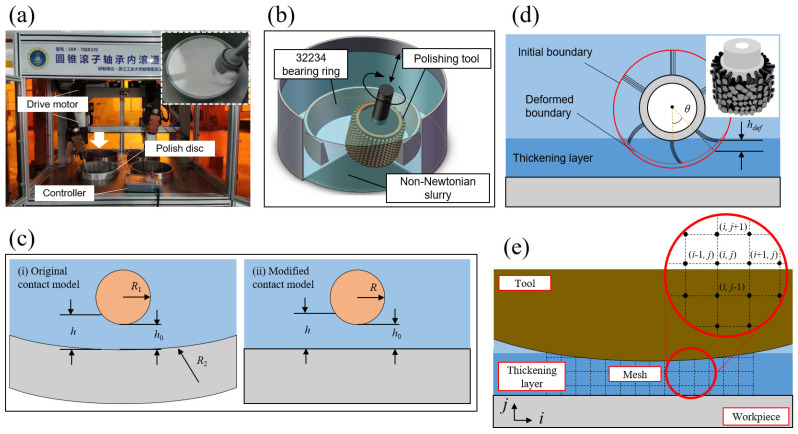
Overview of the manufacturing system: (**a**) prototype machine, (**b**) schematic of the polishing process, (**c**) computational domain boundaries, (**d**) simplification of the polishing model, and (**e**) illustration of mesh grid division.

In the research, the workpiece is the 32,234 cone roller bearing outer race, and the polishing tool selected is a flexible fiber to prevent damage to the workpiece. The experiment was conducted on the multi-axis bearing raceway polishing machine SRP370, as shown in Figure 4a. The polishing machine features bidirectional translation along the x and y axes, with the y-axis spindle positioned at the center of the workpiece. Both the polishing tool (Axis A) and the workpiece (Axis B) exhibit rotational motion and can achieve multi-axis linkage with the x and y spindles. The maximum rotational speed of Axis A is 300 rpm, and its rigidity ensures that the gap h along the raceway axis is uniform. Axis B has a maximum rotational speed of 100 rpm, while the maximum translation speed of the x and y spindles is 2000 mm/s. Figure 4b shows a schematic of the 32,234 polishing process, and the image in the upper-right corner of Figure 4a depicts the actual processing of the outer race, which is clamped in place by a fixture. Surface profiles and roughness were measured using the Taylor PGI810 surface profilometer (Taylor Hobson Ltd., Leicester, UK) and the Mar GD120 surface profilometer (Mahr GmbH, Göttingen, Germany).

## 3. Simulation and Analysis

### 3.1. Simplification of Geometric Models

In practical polishing processes, the polishing tool, which rotates around its axis, typically has a cylindrical cross-section, while the surface of the workpiece may have various curved shapes. Since the contact area width is much smaller than the curvature radius of the contact point, a geometric simplification of the contact surface is possible.

As shown in Figure 4c(i), $R_{1}$ represents the radius of the polishing head, and $R_{2}$ represents the curvature radius of the inner surface of the workpiece. The thickness of the polishing fluid film h can be expressed as follows:

(2) $$h=h_{0}+(R_{1}-\sqrt{R_{1}^{2}-x^{2}})+(R_{2}-\sqrt{R_{2}^{2}-x^{2}})$$

In the equation, $h_{0}$ represents the minimum gap between the tool and the surface. It is important to note that the size of the contact area is much smaller than both the radius of the tool and the curvature radius of the surface, i.e., $\;{x\ll R}_{i}$. Therefore, Equation (2) can be rewritten as follows:

(3) $$h=h_{0}+R_{1}{\left(1-{\frac{x}{R_{1}}}^{2}\right)}^{\frac{1}{2}}+{R_{2}(1-{\frac{x}{R_{2}}}^{2})}^{\frac{1}{2}}$$

Expanding the term ${\left(1-{\frac{x}{R_{i}}}^{2}\right)}^{\frac{1}{2}}$ using a Taylor series and neglecting higher-order small terms such as $\frac{x}{R_{i}}$, the following expression can be derived:

(4) $$h=h_{0}+\frac{x^{2}}{2R^{\prime }}$$

In the equation, $R^{\prime }=\frac{R_{1}R_{2}}{R_{2}-R_{1}}$ represents the equivalent radius of the geometric model, where $R_{2}>R_{1}$.

Therefore, the geometric model is adjusted from the initial hyperboloid model shown in Figure 4c(i) to an equivalent cylindrical and planar contact model, as depicted in Figure 4c(ii).

The thickness h calculated above determines the initial position of the polishing tool. Due to the influence of the slurry’s dynamic-viscous properties during the machining process, the boundary of the polishing tool, $B_{\left(x,y\right)}$, undergoes deformation (as shown in Figure 4d). The actual boundary of the polishing tool, $B_{\left(x,y\right)}$, can be expressed as follows:

(5) $$B_{\left(x,y\right)}=B_{ini}+B_{def}$$

Here, $B_{ini}$ represents the boundary of the tool when both the tool and the workpiece are stationary, as determined by the aforementioned thickness h. $B_{def}$ represents the deformation caused by external forces. The deformation of the flexible fibers under shear force can be described by Equation (6):

(6) $$w_{B}=\int_{0}^{lsin\theta }\frac{pl^{4}}{8EI_{y}}d_{x}$$

In this equation, p is the load applied to the bristles, which depends on the spatial position x; l is the length of the bristles; E is the Young’s modulus of the bristle fibers; and $I_{y}$ is the moment of inertia of the bristles about the y-axis.

The physicochemical properties and structural dimensions of the polishing tool are shown in Table 1. According to previous studies [38], the maximum pressure at the center is on the order of 10^5^ Pa. By inputting the pressure magnitude, structural dimensions, and material properties of the polishing tool into Equation (6), the elastic deformation of the polishing tool is calculated to be on the order of 0.09 mm. Considering the characteristic dimensions on the millimeter scale, this deformation is negligible. Therefore, the polishing tool can be considered to undergo no deformation.

### 3.2. Velocity Distribution

**Figure 5:**
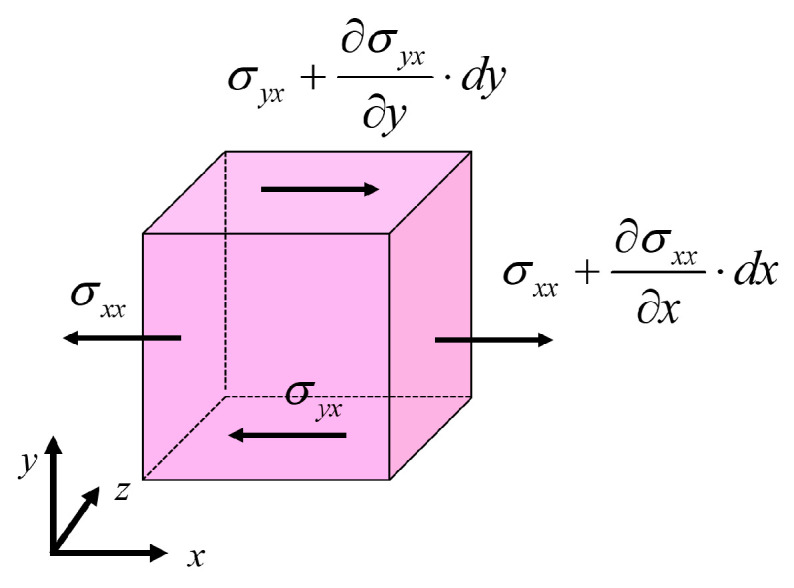
Schematic representation of the microscopic force acting on the slurry.

According to assumption (3), since inertial forces are neglected, the force balance equation shown in Figure 5 should have zero on the right-hand side:

(7) $$\frac{\partial \sigma_{xx}}{\partial x}+\frac{\partial \sigma_{yx}}{\partial y}=0$$

In Equation (7), the fluid element is subjected only to normal pressure and viscous forces between the infinitesimal volumes. Therefore, the normal stress $\sigma_{xx}$ is the negative of the pressure p (with compressive stress as negative and tensile stress as positive), while $\sigma_{yx}$ represents the shear stress τ between adjacent fluid elements. Hence, Equation (7) can be rewritten as follows:

(8) $$-\frac{\partial p}{\partial x}+\frac{\partial \tau }{\partial y}=0$$

Based on assumption (6), it can be concluded that:

(9) $$\frac{\partial p}{\partial y}=0$$

Substituting the constitutive equation, Equation (1), into Equation (8):

(10) $$\frac{1}{\mu_{0}}\frac{\partial p}{\partial x}=\frac{\partial }{\partial y}{\left(\frac{\partial u}{\partial y}\right)}^{n}$$

Considering that p is a function of x only, integrating both sides of Equation (10) gives the following equation:

(11) $${\left(\frac{\partial u}{\partial y}\right)}^{n}=\int \frac{1}{\mu_{0}}\frac{\partial p}{\partial x}d_{y}+C_{1}$$

Integrating the above equation once more with respect to y:

(12) $$u=\int {\left(\frac{y}{\mu_{0}}\frac{\partial p}{\partial x}d_{y}+c_{1}\right)}^{\frac{1}{n}}d_{y}+C_{2}$$

This results in:

(13) $$u={\frac{\mu_{0}}{\frac{\partial p}{\partial x}}\frac{n}{n+1}(\frac{y}{\mu_{0}}\frac{\partial p}{\partial x}d_{y}+c_{1})}^{\frac{n}{n+1}}+C_{2}$$

Introducing the no-slip boundary condition at the wall, the velocity distribution boundary conditions at the workpiece surface and the tool surface are as follows:

(14) $$\left\{\begin{array}{c} y=0,u=0\\ y=h,u=U_{t} \end{array}\right.$$

Here, $U_{t}$ is the velocity at the edge of the polishing tool.

Theoretically, substituting the boundary condition in Equation (14) into the velocity function, the constants $C_{1}$ and $C_{2}$ can be determined. However, an analytical solution for Equation (13) is difficult to obtain. In practice, although $C_{1}$ and $C_{2}$ are independent of y, they are functions of x. If an analytical solution for $C_{1}$ and $C_{2}$ in symbolic form cannot be found, it becomes challenging to obtain accurate results. Huang et al. [39,40] proposed a method for velocity separation. The theory is to decompose the velocity of a non-Newtonian fluid into two components: the shear velocity component $u_{1}$ (hereinafter referred to as “shear velocity”), which changes due to the velocity difference between the upper and lower boundaries, and the pressure velocity component $u_{2}$ (hereinafter referred to as “pressure velocity”), which changes due to variations in pressure.

The shear velocity is only related to the velocity difference between the upper and lower boundaries (i.e., $U_{t}$), so the partial derivative with respect to pressure is zero:

(15) $$\frac{\partial p}{\partial x}=\frac{\partial }{\partial y}(g(\frac{\partial u_{1}}{\partial y}))=0$$

In the above equations: g represents the function of the constitutive equation. Equation (15) indicates that the shear velocity distribution function is independent of y:

(16) $$g\left(\frac{\partial u_{1}}{\partial y}\right)=const(x)$$

In this equation, const(x) represents a constant term that may depend on the function of x. Since the velocity gradient function of the shear rate is independent of y, its inverse function must also be a constant term that does not depend on y. Therefore, the integral of the velocity gradient can be expressed as follows:

(17) $$u_{1}=c_{1}y+c_{2}$$

The expression above must also satisfy the boundary conditions presented in Equation (14), allowing for the determination of the constant term:

(18) $$u_{1}=U_{t}\frac{y}{h}$$

To solve for the pressure velocity distribution, the constitutive equation is substituted into Equation (13) and integrated with respect to y. It is important to note that the pressure-driven flow exhibits symmetry:

(19) $$\frac{\partial u_{2}}{\partial y}={\left(\frac{1}{\mu_{0}}\frac{\partial p}{\partial x}(y-\frac{h}{2})\right)}^{\frac{1}{n}}$$

Integrating the above equation and applying the wall boundary conditions allows the determination of the constant of integration, resulting in the velocity distribution:

(20) $$u_{2}=\frac{n}{n+1}{\left(\frac{1}{\mu_{0}}\frac{\partial p}{\partial x}\right)}^{\frac{1}{n}}({\left(y-\frac{h}{2}\right)}^{1+\frac{1}{n}\;}-{\left(\frac{h}{2}\right)}^{1+\frac{1}{n}})$$

By superimposing Equations (18) and (20), the velocity distribution of the slurry within the computational domain is obtained as follows:

(21) $$u=u_{1}+u_{2}=U_{t}\frac{y}{h}+\frac{n}{n+1}{\left(\frac{1}{\mu_{0}}\frac{\partial p}{\partial x}\right)}^{\frac{1}{n}}({\left|y-\frac{h}{2}\right|}^{1+\frac{1}{n}}-{\left(\frac{h}{2}\right)}^{1+\frac{1}{n}})$$

Considering the nonlinearity introduced by the thickening exponent, the sign of Equation (21) needs to be adjusted to ensure the logical accuracy of the numerical calculations and avoid the appearance of imaginary components:

(22) $$u=U_{t}\frac{y}{h}+sign(\frac{\partial p}{\partial x})\frac{n}{n+1}{\left|\frac{1}{\mu_{0}}\frac{\partial p}{\partial x}\right|}^{\frac{1}{n}}({\left|y-\frac{h}{2}\right|}^{1+\frac{1}{n}}-{\left(\frac{h}{2}\right)}^{1+\frac{1}{n}})$$

Here, $sign(\frac{\partial p}{\partial x})$ represents the sign of $\frac{\partial p}{\partial x}$, indicating whether it is positive or negative.

### 3.3. Pressure Distribution

By substituting Equation (21) into the continuity equation and integrating it using the flow continuity condition, an equation with a separable partial derivative term for pressure can be obtained [41,42]. Upon separating the variables, Equation (23) is derived:

(23) $$\frac{d_{p}}{d_{x}}=sign|h-\bar{h}|\frac{2\mu_{0}}{h^{2n+1}}U_{t}^{n}{\left(2\left(2+\frac{1}{n}\right)\right)}^{n}{\left|h-\bar{h}\right|}^{n}$$

Here, $sign\left(h-\bar{h}\right)$ represents the sign of $\left(h-\bar{h}\right)$, indicating whether it is positive or negative.

It is important to note that when the material of the polishing tool is changed, the deformation may become substantial enough that it can no longer be ignored. Consequently, the form of Equation (23) should be adjusted as follows:

(24) $$\frac{d_{p}}{d_{x}}=f\left(p,w_{B},x\right)=f\left(p,x\right)$$

Equation (24) indicates that the distribution of the boundary h is a function of pressure p and spatial position x, with pressure p (including velocity distribution u) being related to h. These two variables require iterative solving. To ensure both computational accuracy and convergence of the nonlinear equations in this case, the implicit RK4 method is used.

In this study, due to the near-rigid characteristics of the polishing tool, the right-hand side of Equations (22) and (23) does not contain differential terms; thus, the Euler finite difference method (Appendix A) is employed. According to the principles of tribology, positive pressure is generated only by flow through a shrinking gap, meaning that material removal occurs solely on the shrinking side. Therefore, the boundary of the computational domain is set to the left edge of the polishing tool’s arc and the minimum gap, i.e., x∈(−R,0). Under the known geometric structure of the model, the boundary conditions can be expressed as follows:

(25) $$p_{x=-R}=0,\;p_{x=R}=0$$

**Figure 6:**
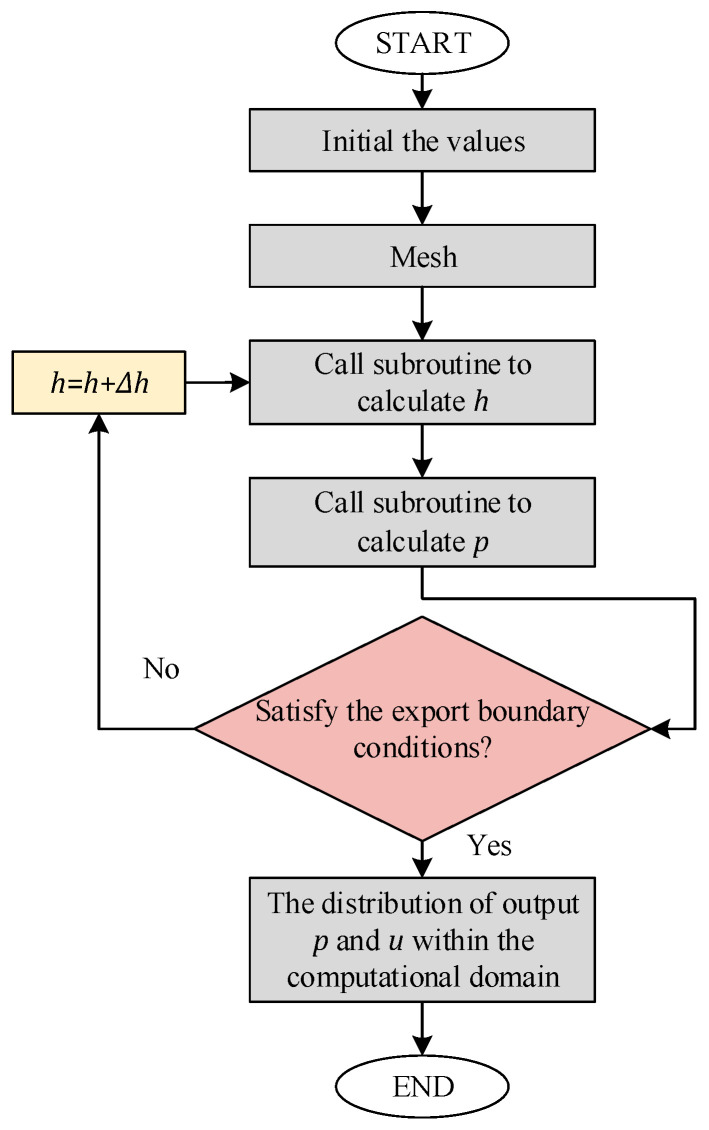
Flowchart of the proposed CFD.

The calculation begins with iteration from both the inlet and outlet. It is important to note that although there is only one unknown variable, p, two boundary conditions are required. This is because Equation (23) is derived through two integrations, and contains an $\bar{h}$, which is unknown. Therefore, $\bar{h}$ must be iteratively corrected during the calculation process, as shown in Figure 6.

The iteration starts with the boundary condition at the inlet, $p_{x=-R}=0$, and an initial estimate for $\bar{h}$ is given (typically the arithmetic mean of the maximum and minimum film thicknesses). Then, $\bar{h}$ is adjusted iteratively to satisfy the outlet condition, $p_{x=R}=0$. Since there is only one zero crossing, the bisection method is used for iteration. Additionally, due to the significant pressure variation near the center, the iteration step size is adjusted accordingly. The iteration proceeds until the calculated outlet pressure falls below 10^−8^, with no upper limit on the number of iterations. The computational domain mesh is shown in Figure 4e, with a grid size of 0.01 × 0.01 mm. Once the pressure distribution is obtained, it is substituted into Equation (22) to determine the velocity distribution.

### 3.4. Removal Model of Single Abrasive

**Figure 7:**
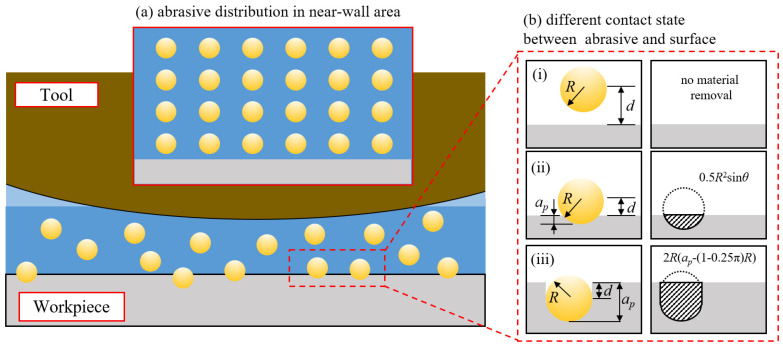
State of abrasives in the near-wall region: (**a**) abrasive distribution assumption and (**b**) abrasive-surface contact state, where: (**i**) d>R & $a_{p}<0$, (**ii**) d<R & $a_{p}>0$, (**iii**) $a_{p}>R$.

After determining the velocity and pressure distributions within the computational domain, it is essential to calculate the number of effective abrasive particles involved in the process. Based on assumption (3), the abrasive particles are uniformly distributed, as illustrated in Figure 7a. Since the experimental system measures the mass concentration of the abrasive particles, it is necessary to establish a relationship between mass and volume concentrations [43] as follows:

(26) $$C_{v}=\frac{\rho_{m}C_{m}}{\rho_{m}C_{m}+\rho_{a}(1-C_{m})}$$

Assuming the abrasive particles are spherical, the number of effective particles per unit area, $N_{a}$, is expressed as follows:

(27) $$N_{a}=\frac{3C_{v}}{4\pi R^{3}}d_{s}$$

In the abrasive polishing process, the contact between the particles and the surface can be classified into three types, as illustrated in Figure 7. When the polishing fluid pressure is insufficient to press the particles into the material surface (d>R), no material removal occurs. When the pressure is sufficient to press the particles to a certain depth (d<R), the projected area of the volume removed by a single particle in the direction of motion is $\frac{1}{2}R^{2}sin\theta$, and the removal efficiency is $\frac{1}{2}R^{2}sin\theta \cdot u$. When the pressure is high enough to embed the geometric center of the particle below the surface ($a_{p}>R$), the projected area of the volume removed by a single particle in the direction of motion is $2R(a_{p}-(1-\frac{\pi }{4})R)$, and the removal efficiency is $2R(a_{p}-(1-\frac{\pi }{4})R)\cdot u$. The depth of particle penetration, $a_{p}$, is determined based on contact mechanics.

(28) $$a_{p}=\sqrt{\frac{2F_{n}}{\pi \sigma_{s}{tan}^{2}\alpha_{c}}}$$

### 3.5. Material Removal Model

Material removal function can be expressed as Equation (29), based on the analyses above:

(29) $$V_{MRR}=\int_{0}^{S}\int_{0}^{T}\frac{3upC_{v}}{4\pi \sigma_{s}Rtan\alpha_{c}}d_{t}d_{s}$$

By substituting the equations for the physical quantities within the computational domain, the complete material removal distribution function is obtained as follows:

(30) $$V_{MRR}=\int_{0}^{T}\int \frac{3upC_{v}}{4\pi \sigma_{s}Rtan\alpha_{c}}\int \frac{2\mu_{0}}{h^{2n+1}}U^{n}{\left(2(2+\frac{1}{n})\right)}^{n}{\left(h-\bar{h}\right)}^{n}d_{t}d_{x}(U_{t}\frac{y}{h}+\frac{n}{1+n}{\left(\frac{1}{\mu_{0}}\frac{\partial p}{\partial x}\right)}^{\frac{1}{n}})({\left|y-\frac{h}{2}\right|}^{\frac{1}{n}+1}-{\left(\frac{h}{2}\right)}^{\frac{1}{n}+1}))d_{s}$$

## 4. Validation and Analysis

### 4.1. Valid Experiment Design

The experiment aims to investigate the effects of different relative velocities (u) and gap distances (h) on material removal, thereby validating the accuracy of the material removal model. A series of single-factor experiments were conducted to assess the uniformity of material removal under varying u and h, with a polishing time of 60 min. The levels of the other process parameters are provided in Table 2.

**Figure 8:**
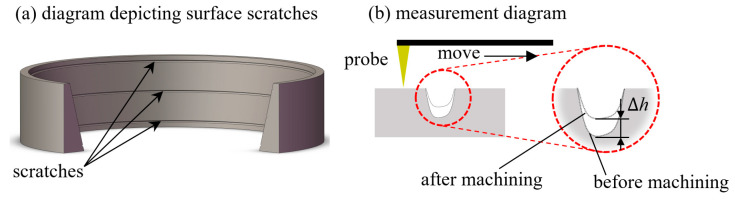
Scratch distribution and measurement: (**a**) scratch schematic and (**b**) scratch measurement.

Due to the large mass of the workpiece and the relatively small amount of material removal, a precision balance cannot simultaneously satisfy both measurement requirements. To accurately quantify material removal during the processing cycle, three evenly spaced scratches were made axially on the outer bearing (32,234) raceway (Figure 8a), corresponding to inner diameters of 285 mm, 270 mm, and 255 mm. The yield strength of the material without heat treatment is 400 MPa. The material removal rate during this period was determined by measuring the change in distance, Δh, between the bottom of the scratches and the lower top surfaces before and after processing (Figure 8b).

### 4.2. Validation Results and Discussion

**Figure 9:**
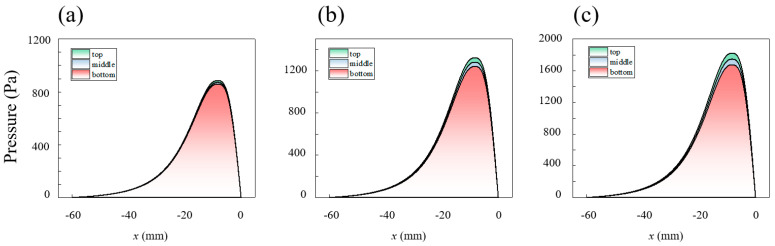
Effect of different velocities on the pressure distribution of the slurry within the computational domain: (**a**) 10 rpm, (**b**) 25 rpm, and (**c**) 40 rpm.

**Figure 10:**
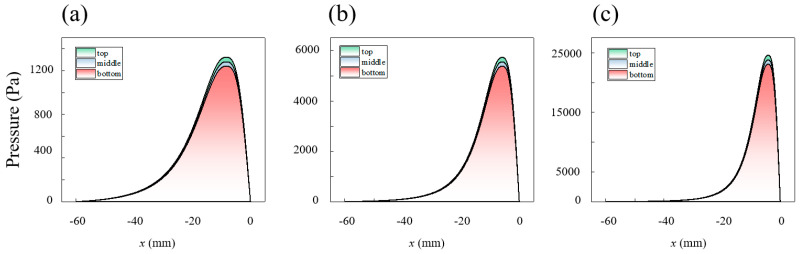
Effect of gap on the pressure distribution of the slurry within the computational domain: (**a**) 2 mm, (**b**) 1 mm, and (**c**) 0.5 mm.

**Figure 11:**
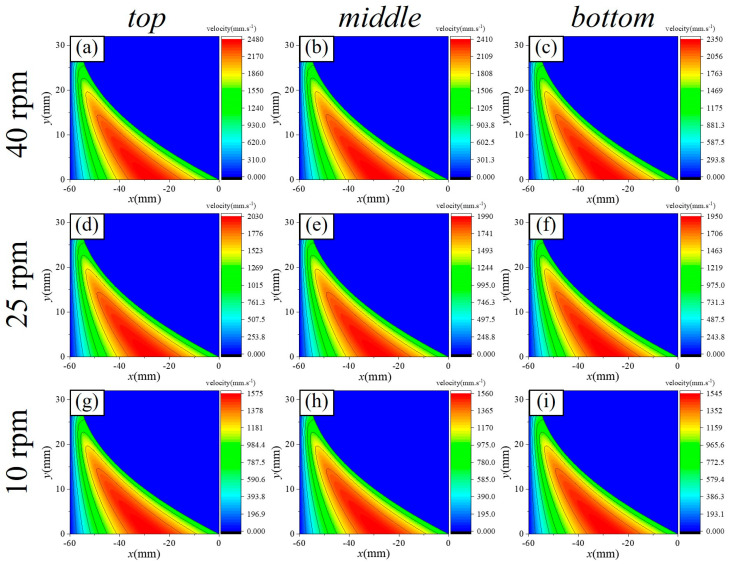
Effect of *U* on the velocity distribution of the slurry within the computational domain. Classified by speed: (**a**–**c**) with a rotation velocity of 40 rpm, (**d**–**f**) with a rotation velocity of 25 rpm, (**g**–**i**) with a rotation velocity of 10 rpm. Classified by position: (**a**,**d**,**g**) on the top of the raceway, (**b**,**e**,**h**) on the top of the raceway, (**c**,**f**,**i**) on the top of the raceway.

**Figure 12:**
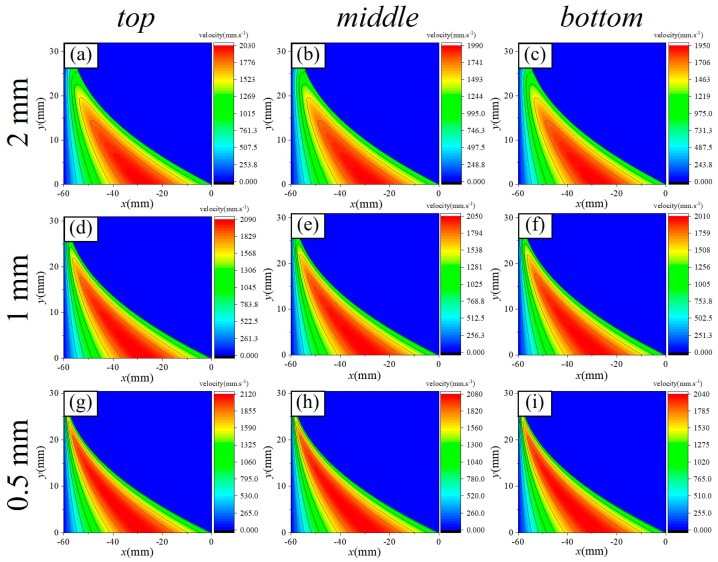
Effect of h on the velocity distribution of the slurry within the computational domain. Classified by speed: (**a**–**c**) with a rotation velocity of 40 rpm, (**d**–**f**) with a rotation velocity of 25 rpm, (**g**–**i**) with a rotation velocity of 10 rpm. Classified by position: (**a**,**d**,**g**) on the top of the raceway, (**b**,**e**,**h**) on the top of the raceway, (**c**,**f**,**i**) on the top of the raceway.

**Figure 13:**
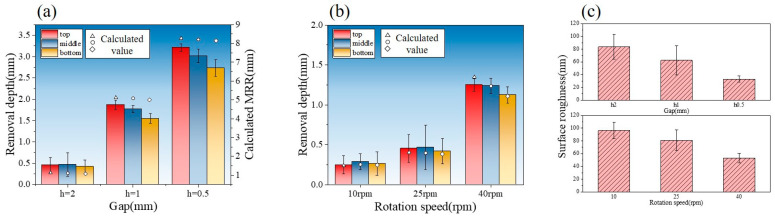
Validation result: (**a**,**b**) comparison of material removal between validation results and theoretical calculations at varying gaps and speeds and (**c**) evaluation of surface roughness under different processing conditions.

Figure 9, Figure 10, Figure 11, Figure 12 and Figure 13 show the pressure and velocity distributions in the effective processing region under different relative speeds and gap sizes. Due to Equation (15) ($\frac{\partial p}{\partial y}=0$), the pressure distribution is presented as a 2D line plot, with green, purple, and red regions representing the pressure distributions at the top, middle, and bottom of the raceway, respectively. The velocity distribution is shown as a contour plot. Unless otherwise specified, the data refers to the position at the top of the raceway.

#### 4.2.1. Analysis of Simulation Results

##### Pressure Distribution Law

The pressure variation in the slurry along the flow direction within the computational domain is shown in Figure 9 ($h_{0}=2\;mm$) and Figure 10 (ω=25 rpm). The pressure jump occurs near the minimum gap (pressure outlet). In comparison to Newtonian fluids or shear-thinning fluids, the peak shape of shear-thickening fluids is much steeper, indicating that the viscosity change enhances the amplitude of the pressure variation [40].

Furthermore, the relationship between pressure distribution, rotational speed, and gap can be summarized as follows:

There is a pressure difference between the upper and lower parts of the raceway, caused by the velocity differences induced by the raceway structure. The pressure is highest at the inner diameter of the raceway top, which results in the pressure distribution being highest at the top, although the difference is small.

The pressure peak difference between different areas and the relative speed are proportional. When the raceway rotational speed is 10 rpm, the maximum pressure is 885.9 Pa, with a pressure difference of 26.9 Pa between the top and bottom. In contrast, when the raceway rotational speed is 40 rpm, the maximum pressure reaches 1821.7 Pa, with a pressure difference of 146.4 Pa between the top and bottom.

As the gap decreases, the pressure peak and the pressure difference at different areas along the raceway increase, and the pressure peak shifts away from the pressure outlet. When the gap is 2 mm, the pressure peak is 1322.6 Pa, occurring 0.84 mm from the outlet, with a pressure difference of 80.7 Pa between the top and bottom. When the gap is 0.5 mm, the pressure peak increases to 24,569.6 Pa, occurring 0.42 mm from the outlet, with a pressure difference of 1498.8 Pa between the top and bottom. The pressure difference at this gap even exceeds the peak pressure at a 2 mm gap.

##### Velocity Distribution Law

The variation in velocity along the flow direction within the computational domain is depicted in Figure 11 ($h_{0}=2\;mm$) and Figure 12 (ω=25 rpm). Figure 11a–c, Figure 11d–f, and Figure 11g–i illustrate the velocity distribution of the slurry within the computational domain at ring rotational speeds of 40 rpm, 25 rpm, and 10 rpm, respectively. Figure 12a–c, Figure 12d–f, and Figure 12g–i show the velocity distribution of the slurry for initial minimum gaps of 2 mm, 1 mm, and 0.5 mm, respectively. The peak velocity of the slurry is observed not at the wall surface, but in the central region of the flow. For material removal calculations, velocity values near the wall must be extracted.

Furthermore, the relationship between the velocity distribution, rotational speed, and gap can be summarized as follows:

(1) At the same rotational speed, varying raceway radii across different regions result in different relative velocities. However, these differences are minimal, with the maximum variation being only 130 mm/s.
(2) The ring rotational speed has the most significant impact on relative velocity. As the ring speed decreases from 40 rpm to 10 rpm, the peak velocity of the slurry decreases by 955 mm/s. Notably, the peak velocity does not occur at the wall but in the central region of the flow. For material removal calculations, velocity values near the wall are necessary.
(3) As the gap decreases, the peak velocity increases; however, this increase is much smaller compared to the change in pressure, as the increase in viscosity hinders slurry motion. When the gap is reduced from 2 mm to 0.5 mm, the peak velocity increases from 2030 mm/s to 2120 mm/s.

In summary, the gap exerts a substantial influence on the pressure distribution of the slurry, with its effect on pressure being an order of magnitude (10^1^ times) greater than that of relative velocity. Moreover, higher rotational speeds lead to increased slurry velocities near the wall. Therefore, processing conditions characterized by small gaps and high relative velocities optimize material removal efficiency.

#### 4.2.2. Validation Result

Figure 13 presents a comparison between the experimental results and theoretical calculations of material removal efficiency under different process parameters. Figure 13a compares the results of the removal model calculations and experiments at ω=25 rpm for different gap sizes. When the minimum gap is 2 mm, the removal thickness of the inner raceway ranges from 0.417 to 0.453 μm, with a theoretical model median of 1.103 μm. At a gap of 1 mm, the removal thickness ranges from 1.549 to 1.872 μm, with a model median of 5.082 μm. For a gap of 0.5 mm, the removal thickness ranges from 2.732 to 3.209 μm, with a model median of 8.214 μm. Due to the assumptions made in the model, deviations from actual processing conditions are inevitable, necessitating model correction. As shown in Figure 13a, the deviation between the theoretical model and experimental results is linear, allowing for the application of a linear correction factor, k. The value of k is determined as the arithmetic mean of all deviations, k=0.361. After correction, the maximum error occurs at the small gap of $h_{0}=0.5\;mm$, at approximately 16.3%, with an average error of about 12.5%.

The comparison between the corrected model’s calculated values and theoretical values at different rotational speeds was also examined. Figure 13b compares the model and experimental values at $h_{0}=2\;mm$ for different rotational speeds. When the ring speed is 10 rpm, the removal thickness of the inner raceway ranges from 0.247 to 0.287 μm, with a model median of 0.237 μm. At 25 rpm, the removal thickness ranges from 0.417 to 0.453 μm, with a model median of 0.397 μm. At 40 rpm, the removal thickness ranges from 1.124 to 1.249 μm, with a model median of 1.346 μm. At higher rotational speeds, the model shows a maximum error of approximately 14.0%, with an average error of about 11.1%.

In summary, the corrected model exhibits a maximum error of no more than 17% and an average error of no more than 13%. The datum is well-distributed and accurate, making it a reliable tool for material removal prediction.

Figure 13c presents a comparison of surface roughness (*Ra*) after polishing under various conditions. As the roughness values at the top, middle, and bottom showed minimal differences, the figure displays the overall average roughness across all positions. The roughness variation follows the same trend as material removal efficiency: higher rotational speeds and smaller gaps lead to the lowest roughness. Therefore, the model-predicted material removal efficiency can serve as an effective indicator for optimizing the process parameters that influence surface quality.

#### 4.2.3. Optimal Experiment

**Figure 14:**
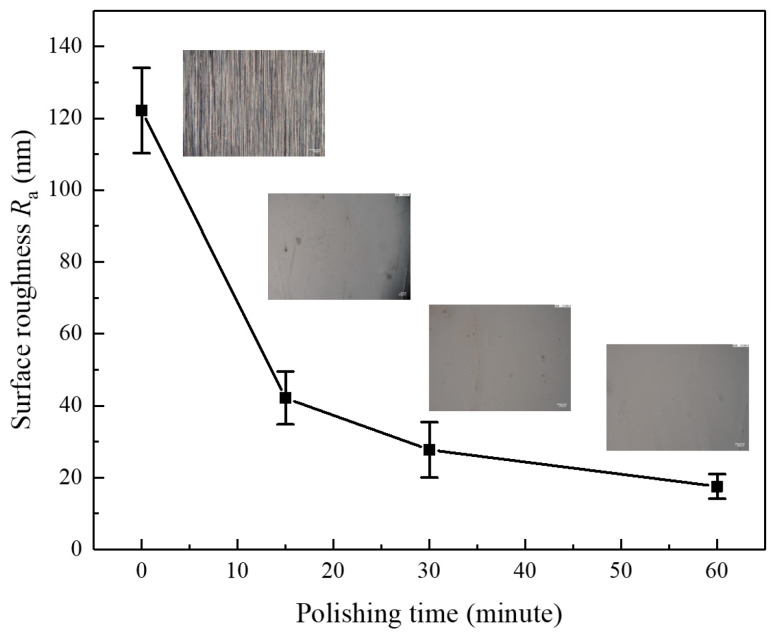
Variation in surface roughness with polishing time.

Based on the analysis and experimental results, 0.5 mm and 40 rpm were identified as the optimal process parameters, with other variables held constant. Figure 14 illustrates the evolution of surface roughness in terms of *Ra* over time under these optimal conditions. After 30 min of polishing, the surface roughness decreased to 27.74 nm, outperforming all results obtained under different parameter settings in the verification tests. After 60 min of polishing, the surface roughness further decreased to 17.59 nm, demonstrating high consistency in surface quality, with the roughness variance of only 4.42 nm^2^.

**Figure 15:**
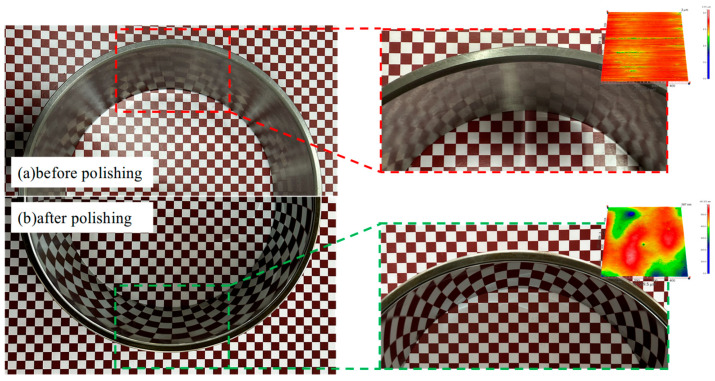
Comparison of surface quality of 32,234 raceways before and after STP, where (**a**) after grinding and (**b**) after STP processing.

Figure 15 provides a comparison of the true picture before and after polishing. Before polishing (Figure 15a), the raceway surface, following precision grinding, exhibited a microstructure with groove-like stripes, and the surface showed a blurred reflective quality. In contrast, after polishing (Figure 15b), the raceway surface was free from visible machining marks, presenting a smooth, bright, mirror-like finish.

## 5. Conclusions

This paper presents an advanced shear-thickening polishing method that applies normal pressure to the workpiece surface assisted by the motion of a tool. A material removal distribution model is developed, with the processing target being the raceway of a large 32,234 bearing outer ring. The model is refined through valid experiments. The main research contents and conclusions are as follows:

(1) The rheological properties of the slurry were measured, and reasonable assumptions were established based on machining conditions and relevant theory.
(2) Distribution functions for pressure and velocity within the processing area were derived. The velocity distribution within the computational domain was obtained using velocity decomposition, then the pressure distribution was computed using the finite difference method.
(3) A single abrasive removal function was established based on contact mechanics, and a material removal distribution function was derived using metallographic analysis.
(4) Valid experiments were carried out under different process parameter levels. The results indicated that the average error of the modified theoretical model was approximately 12%, with a maximum error of 16.3%. The model demonstrated high accuracy and high reliability.
(5) The surface roughness and material removal rate exhibited similar trends, allowing the removal rate to serve as an indicator for identifying the optimal process parameters for achieving the best surface quality. The optimization experiment yielded a surface with a roughness of *Ra* 17.59 nm and a variance of 4.42 nm^2^, demonstrating good processing consistency.

This research presented in this paper aims to provide reliable material removal predictions for the rheological flexible polishing process, offering significant engineering application value.

## Figures and Tables

**Table 1:** Properties and structural dimensions of polishing tool [39].

Tool Parameter	Value
Material	nylon fiber
Young’s modulus	5 Gpa
Fiber diameter	0.5 mm
Fiber length	10 mm

**Table 2:** Process parameters for validation experiments.

Processing Parameters	Value
Rotation speed of workpiece (rpm)	10, 25, 40
Inner diameter of workpiece (mm)	252~298
Rotation speed of tool (rpm)	100
Diameter of tool (mm)	60
Gap *h* (mm)	0.5, 1, 2

## Data Availability

The datasets generated and/or analyzed during the current study are not publicly available due to the laboratory policy but are available from the corresponding author on reasonable request.

## Nomenclature

Nomenclature		$a_{p}$	depth of particle penetration
B	actual boundary	$B_{ini}$	boundary without deformance
$B_{def}$	deformation of the tool	$C_{v}$	volume concentrations
E	tool’s Young’s modulus	F	force on the tool
$F_{n}$	normal component of F	$F_{t}$	tangential component of F
h	gap size	$h_{0}$	minimum film thickness
$I_{y}$	inertia moment	k	correction factor
l	fiber length	n	thickening index
$N_{a}$	number of active abrasive	p	slurry pressure
R	equivalent radius	$R_{1}$	tool radius
$R_{2}$	workpiece radius	T	residence time
$U_{h}$	upper boundary rate	$U_{0}$	lower boundary rate
u	rate component in the x-direction	v	rate component in the y-direction
$V_{MRR}$	material removal rate	$w_{B}$	nylon deflection
δ	film thickness	θ	the central angle corresponding to the interval between different clusters of bristles
μ	viscosity	$\mu^{*}$	apparent viscosity
ρ	density		

## Appendix A. Euler’s Difference Method and Its Implementation

The differential equation is expressed in the form, as shown in Equation (A1):

(A1) $$y^{\prime }=f(x,y)$$

The Euler forward finite difference method approximates the differential value using the curvature at a given point, which can be expressed as follows:

(A2) $$y^{\prime }(x_{n+1})=\frac{y\left(x_{n+1}\right)-y\left(x_{n}\right)}{x_{n+1}-x_{n}}$$

where $y^{\prime }(x_{n+1})$ is the differential value of the function at $\;x_{n+1}$,

$y\left(x_{n+1}\right)$ is the value of the function at $x_{n+1}$,

$y\left(x_{n}\right)$ is the value of the function at $x_{n}$,

$x_{n+1}-x_{n}$ is the step size, denoted as ∆x in subsequent expressions.

Therefore, the function value at the next grid point can be expressed as follows:

(A3) $$y\left(x_{n+1}\right)=y\left(x_{n}\right)+(x_{n+1}-x_{n})f(x,y)$$

Taking the calculation of pressure distribution as an example: discretizing Equation (A3) using the finite difference method and substituting the initial pressure values, the pressure distribution can be calculated using Equation (A4) as follows:

(A4) $$p_{i+1}=p_{i}+∆x\cdot f(x,y)$$

Performing a Taylor series expansion of $y\left(x_{n+1}\right)$ around $x_{n}$, the function can be expressed as follows:

(A5) $$y\left(x_{n+1}\right)=y\left(x_{n}\right)+∆x\cdot y^{\prime }+ο(∆x)$$

Thus, the accuracy of the Euler forward finite difference method is of the order ∆x, making it less suitable for nonlinear, non-homogeneous, or more complex differential equations. In such cases, improved Euler methods or other higher-order methods should be used. The code for the Euler forward finite difference method is not presented here due to its simplicity.
